# MIR155HG Plays a Bivalent Role in Regulating Innate Antiviral Immunity by Encoding Long Noncoding RNA-155 and microRNA-155-5p

**DOI:** 10.1128/mbio.02510-22

**Published:** 2022-11-02

**Authors:** Kul Raj Rai, Yuan Liao, Mengjuan Cai, Haori Qiu, Faxin Wen, Min Peng, Song Wang, Shasha Liu, Guijie Guo, Xiaojuan Chi, Mohamed Maarouf, Yuhai Chen, Shile Huang, Ji-Long Chen

**Affiliations:** a Key Laboratory of Animal Pathogen Infection and Immunology of Fujian Province, College of Animal Sciences, Fujian Agriculture and Forestry University, Fuzhou, China; b CAS Key Laboratory of Pathogenic Microbiology and Immunology, Institute of Microbiology, Chinese Academy of Sciences, Beijing, China; c Department of Biochemistry and Molecular Biology, Louisiana State University Health Sciences Center, Shreveport, Louisiana, USA; Duke University Medical Center; McMaster University

**Keywords:** *MIR155HG*, lncRNA-155, miRNA-155-5p, influenza A virus, STAT1, immune response

## Abstract

*MIR155HG* encodes a precursor RNA of microRNA-155 (miRNA-155). We previously identified this RNA also as a long noncoding RNA (lncRNA) that we call lncRNA-155. To define the functions of miRNA-155 and lncRNA-155, we generated miRNA-155 knockout (KO) mice lacking only 19 bp of the miRNA-155 core sequence without affecting the expression of lncRNA-155. Surprisingly, compared with the miRNA-155KO mice, previously generated lncRNA-155KO mice were more susceptible to both influenza virus (RNA virus) and pseudorabies virus (DNA virus) infection, as characterized by lower survival rate, higher body weight loss, and higher viral load. We found that miRNA-155-5p enhanced antiviral responses by positively regulating activation of signal transducer and activator of transcription 1 (STAT1), but the STAT1 activity differed greatly in the animals (lncRNA-155KO < miRNA-155KO < wild type). In line with this, expression levels of several critical interferon-stimulated genes (ISGs) were also significantly different (lncRNA-155KO < miRNA-155KO < wild type). We found that lncRNA-155 augmented interferon beta (IFN-β) production during the viral infection, but miRNA-155 had no significant effect on the virus-induced IFN-β expression. Furthermore, we observed that lncRNA-155 loss in mice resulted in dramatic inhibition of virus-induced activation of interferon regulatory factor 3 compared to both miRNA-155KO and wild-type (WT) animals. Moreover, lncRNA-155 still significantly suppressed the viral infection even though the miRNA-155 derived from lncRNA-155 was deleted or blocked. These results reveal that lncRNA-155 and miRNA-155 regulate antiviral responses through distinct mechanisms, indicating a bivalent role for *MIR155HG* in innate immunity.

## INTRODUCTION

The human genome is actively transcribed into various RNAs. However, the vast majority (~98%) of transcripts are noncoding RNAs, including microRNAs (miRNAs) and long noncoding RNAs (lncRNAs). Increasing evidence reveals that both microRNAs and lncRNAs regulate multiple cellular processes, including viral pathogenesis, by diverse mechanisms. Some lncRNAs contain microRNA precursor sequences that give rise to functional miRNAs and may also encode micropeptides (1). It is well known that the microRNA (miR)-155 host gene (*MIR155HG*) encodes a critical microRNA, miRNA-155 (2). Importantly, we have recently identified that the precursor sequence of miRNA-155 encoded by *MIR155HG* also acts as an lncRNA, named lncRNA-155 (3) and also known as BIC (4). The miRNA-155 has been well characterized as a master regulator of inflammatory responses (5–7), and the functional relevance of miRNA-155 has been shown in the pathogenesis of several viral infections, including influenza A virus (IAV) (8–11). However, little is known about the implication of lncRNA-155 in the biological processes without processing miRNA-155. Interestingly, the precursor sequence of lncRNA-155 has also been reported to encode a micro-peptide that possesses the ability to modulate the inflammatory response (12). Moreover, lncRNA-155 can also sponge other miRNAs involved in regulating inflammatory responses (13–15). For instance, lncRNA-155 upregulates myocyte enhancer factor 2A (MEF2A) by sponging miR-194-5p (15). Deletion of MEF2A prevents the induction of interferon beta (IFN-β) upon exposure to immune stimuli or pathogens (16), indicating a critical role for lncRNA-155 in the regulation of some biological processes, including innate immunity far behind processing miRNA-155.

Innate immunity is the first line of defense against invading viruses (17). The IFN signaling regulates antiviral innate immune responses, which plays a vital role in viral clearance (18). IFN-mediated innate immune responses are initiated upon sensing of viral components by innate receptors. Interferon regulatory factors 3/7 (IRF3/7) and nuclear factor kappa B (NF-κB) are the main transcriptional factors that govern the expression of type I IFNs (IFN-α/β) (19). The secreted IFNs bind to their respective receptors and activate the Janus kinase-signal transducer and activator of transcription (JAK-STAT) pathway, resulting in the production of many downstream antiviral interferon-stimulating genes (ISGs), which establishes an antiviral state to impede viral infection (20). The activation of IFN signaling is tightly regulated through the JAK-STAT1 pathway, whose dysregulation is associated with various immune disorders and diseases (17, 21, 22). Innate immune signaling triggered by viral infection also induces differential expressions of lncRNAs. These differentially expressed lncRNAs, in turn, can modulate antiviral responses in multiple ways (23, 24) and can also provide a feedback loop to regulate antiviral immune signaling pathways (25, 26). For example, lncLrrc55-AS and lncRNA-PLACT1 provide a positive feedback loop to sustain IRF3 and NF-κB signaling, respectively (27, 28). While the functions of various signaling molecules in these pathways have been well studied, the role of lncRNAs in the regulation of the IFN response and JAK-STAT1 signaling during viral infection remains elusive.

Recently, we have shown that lncRNA-155 is an inducible host lncRNA by IAV infection in human and mouse cells, and the expression of lncRNA-155 is regulated by the retinoic acid-inducible gene I (RIG-I)- and toll-like receptor 3 (TLR3)-dependent innate immune signaling (3). Furthermore, lncRNA-155 can positively regulate innate immunity. On the other hand, miRNA-155 is one of the best-characterized miRNAs as a master regulator of inflammatory responses (5, 29, 30). Of interest, miRNA-155 has both proinflammatory and anti-inflammatory roles by targeting different RNA molecules involved in immune signaling (7, 31). It has been demonstrated that infection of many viruses induces the expression of miRNA-155 that can, in turn, regulate viral replication (8–10, 32).

Although both lncRNA-155 and miRNA-155 have been implicated in antiviral responses to IAV infection (3), little information is available about the functions of lncRNA-155 and miRNA-155 in the pathogenesis of pseudorabies virus (PRV), an important DNA virus. Pseudorabies (also known as Aujeszky’s disease) is a severe infectious disease of pigs caused by PRV, leading to heavy economic losses to the swine industry in many countries (33). In addition, PRV might have the potential risk of human infection, which threatens public health security (34). Currently, vaccination is one of the most widely used strategies to control PRV infection in the swine industry. However, new variants of PRV have emerged in vaccinated pigs in many provinces in China since 2011 (34). This creates the necessity to develop alternative immunotherapeutic approaches for controlling pseudorabies. An understanding of the implication of noncoding RNAs in PRV pathogenesis is essential to explore possible therapeutic approaches against PRV.

The type I IFN response is a critical process to elicit antiviral immunity. Although both lncRNA-155 and miRNA-155 have been implicated in regulating innate antiviral immunity, the resultant effect of the miRNA-155-mediated type I IFN response remains to be determined owing to discrepancies in previous reports. The molecular mechanism of miRNA-155-mediated regulation of the type I IFN response and IFN expression is complex and depends on many factors, such as host species, virus types, and cell types. On the other hand, several molecules involved in antiviral immune signaling have been reported to be targeted by miRNA-155 (6, 35). For example, suppressor of cytokine signaling 1 (SOCS1) has been shown as a main target of miRNA-155 in regulating type I IFN signaling (9, 10). However, it is unclear whether type I IFN signaling is mediated by miRNA-155 alone or also involves lncRNA-155, because several viruses and other stimuli also induce the robust expression of lncRNA-155 along with miRNA-155, and the expression of lncRNA-155 without processing miRNA-155 can also regulate innate immunity (3). More importantly, without appropriate *in vivo* systems, it is difficult to determine the differential impacts of miRNA-155 and lncRNA-155 on the antiviral responses.

In this study, we generated miRNA-155 knockout (KO) mice lacking only 19 bp of the miRNA-155 core sequence and took advantage of miRNA-155KO and lncRNA-155KO mouse models to determine the functional involvement of miRNA-155 and lncRNA-155 in antiviral responses. We observed that inducible lncRNA-155 alone augments IFN production at least by regulating the activation of IRF3, while virus-induced miRNA-155-5p potentiates antiviral responses by positively regulating the activation of STAT1. Our study unravels the distinct immune regulatory mechanisms of lncRNA-155 and miR155-5p.

## RESULTS

### LncRNA-155KO mice are more susceptible to IAV infection than miRNA-155KO and WT mice.

**FIG 1:**
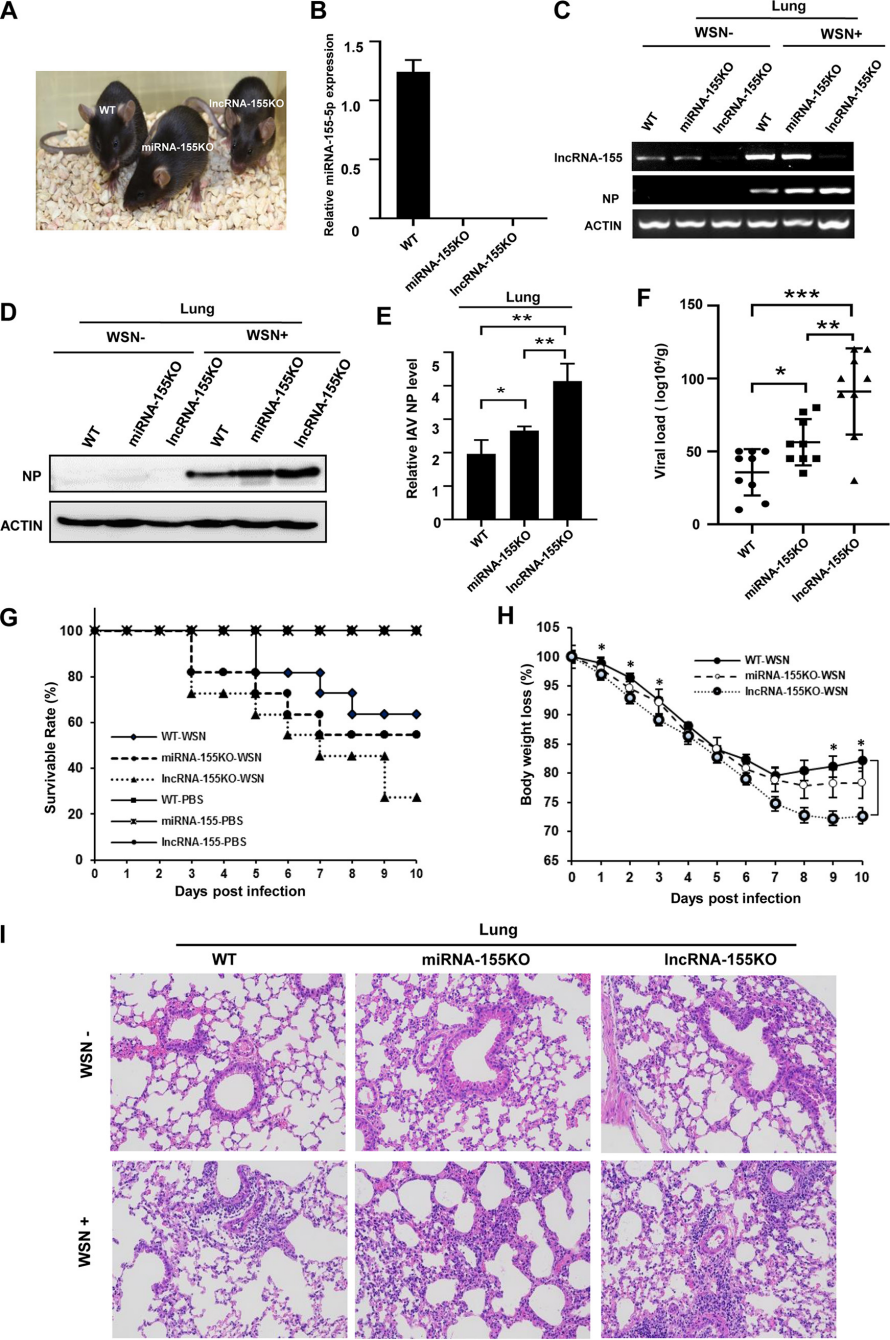
LncRNA-155KO mice are more susceptible to IAV infection than miRNA-155KO and WT mice. (A and B) MiRNA-155KO mice were generated. Shown are representative photographs of wild-type (WT), miRNA-155 knockout (miRNA-155KO), and lncRNA-155 knockout (lncRNA-155KO) mice (A). The expression levels of miRNA-155-5p in the lung tissues of the indicated mice were examined by qRT-PCR (B). (C) The expression levels of lncRNA-155 in lung tissues of the indicated mice with and without influenza virus infection were examined by RT-PCR. (D to I) WT, miRNA-155KO, and lncRNA-155KO mice (5 to 6 weeks old) were intranasally inoculated with 5 × 10^5^ PFU WSN, and the viral virulence and infection kinetics in mice were determined by Western blotting of viral NP protein levels (D and E), measuring viral loads in lungs employing a plaque-forming assay at the indicated dpi (3 to 5 mice/group) (F), survival rates (11 to 13 mice/group) (G), body weight loss (11 to 13 mice/group) (H), and HE staining analysis of mouse lung tissues at 2 dpi (magnification ×200) (I). Data are shown as means ± standard deviation (SD); *, *P < *0.05; **, *P < *0.01; ***, *P < *0.001; *n* = 3.

Our previous experiments have demonstrated that lncRNA-155 is an inducible lncRNA by IAV infection and positively regulates antiviral immunity without miRNA-155 processing (3). The conclusion drawn in the previous study was based on *in vitro* analysis using several cell lines and lncRNA-155KO mice lacking 0.9 kb of genomic sequences that contain both lncRNA-155 and miRNA-155. To determine whether lncRNA-155 and miRNA-155 have different roles in regulating antiviral immunity, we generated miRNA-155KO mice that lack only 19 bp of the miRNA-155 core sequence and maintain the rest sequences (encoding lncRNA-155) intact (Fig. 1A and Fig. S1A in the supplemental material). Knockout of miRNA-155 was confirmed by sequencing (Fig. S1B). The expression of miRNA-155-5p and lncRNA-155 was examined in lung tissues of wild-type (WT), miRNA-155KO, and lncRNA-155KO mice. As expected, only WT mice exhibited the expression of miRNA-155-5p (Fig. 1B). In contrast, comparable levels of lncRNA-155 were observed between miRNA-155KO and WT mice (Fig. 1C and Fig. S1C), indicating that deletion of the 19 bp of the miRNA-155 core sequence had no effect on the expression of lncRNA-155 in mice.

10.1128/mbio.02510-22.1 FIG S1 Generation of miRNA-155 knockout mice and infection of WT, miRNA-155KO, and lncRNA-KO mice with IAV. (A and B) Schematic representation of CRISPR/Cas9-mediated generation of miRNA-155KO mice. The 19 bp of the miRNA-155 core sequence is highlighted in red (A), and the deletion was verified by sequencing analysis (B). (C) RT-PCR analysis of lncRNA-155 expression in WT and miRNA-155KO mice with and without IAV infection. Download FIG S1, TIF file, 0.9 MB. Copyright © 2022 Rai et al. 2022 Rai et al. https://creativecommons.org/licenses/by/4.0/ This content is distributed under the terms of the Creative Commons Attribution 4.0 International license.

Next, the lncRNA-155KO, miRNA-155KO, and WT mice were utilized for *in vivo* experiments in this study. All three groups of mice (*n* = 11 to 13 per group) were infected with A/WSN/33 (H1N1) influenza virus (WSN) intranasally and subjected to susceptibility testing after the IAV infection. Reverse transcriptase PCR (RT-PCR) and Western blotting of viral nucleoprotein (NP) in lung tissues showed that lncRNA-155KO mice had significantly higher levels of viral NP mRNA and protein than those in miRNA-155KO and WT animals at 3 days postinfection (dpi) (Fig. 1C to E). Then, we examined the IAV load in lungs after viral infection by plaque forming assay. Consistently, lncRNA-155KO mice displayed a significant increase of viral loads in the lungs compared to miRNA-155KO and WT mice (Fig. 1F). In line with these results, the survival rate of lncRNA-155KO mice was lower than that of miRNA-155KO or WT mice under our experimental conditions. Both lncRNA-155KO and miRNA-155KO mice began dying as early as three dpi. At 10 dpi, the survival rates of WT, miRNA-155KO, and lncRNA-155KO mice were 63.63%, 54.54%, and 27.27% respectively (Fig. 1G). Supporting this, lncRNA-155KO mice lost body weight more significantly than WT mice following the indicated number of days postinfection (Fig. 1H). Body weight loss of miRNA-155KO mice was higher than that of WT mice but lower than that of lncRNA-155KO mice (Fig. 1H). Furthermore, pathological examination using hematoxylin and eosin (HE) staining showed that lncRNA-155KO mice exhibited more severe inflammation and increased inflammatory cell infiltration in the lungs than miRNA-155KO and WT mice challenged with WSN (Fig. 1I). These data reveal that lncRNA-155KO mice are the most susceptible to IAV infection among these mice, suggesting that lncRNA-155 may have other functions in antiviral responses in addition to processing miRNA-155.

### PRV infection induces robust expression of miRNA-155-5p and lncRNA-155 both *in vitro* and *in vivo*.

**FIG 2:**
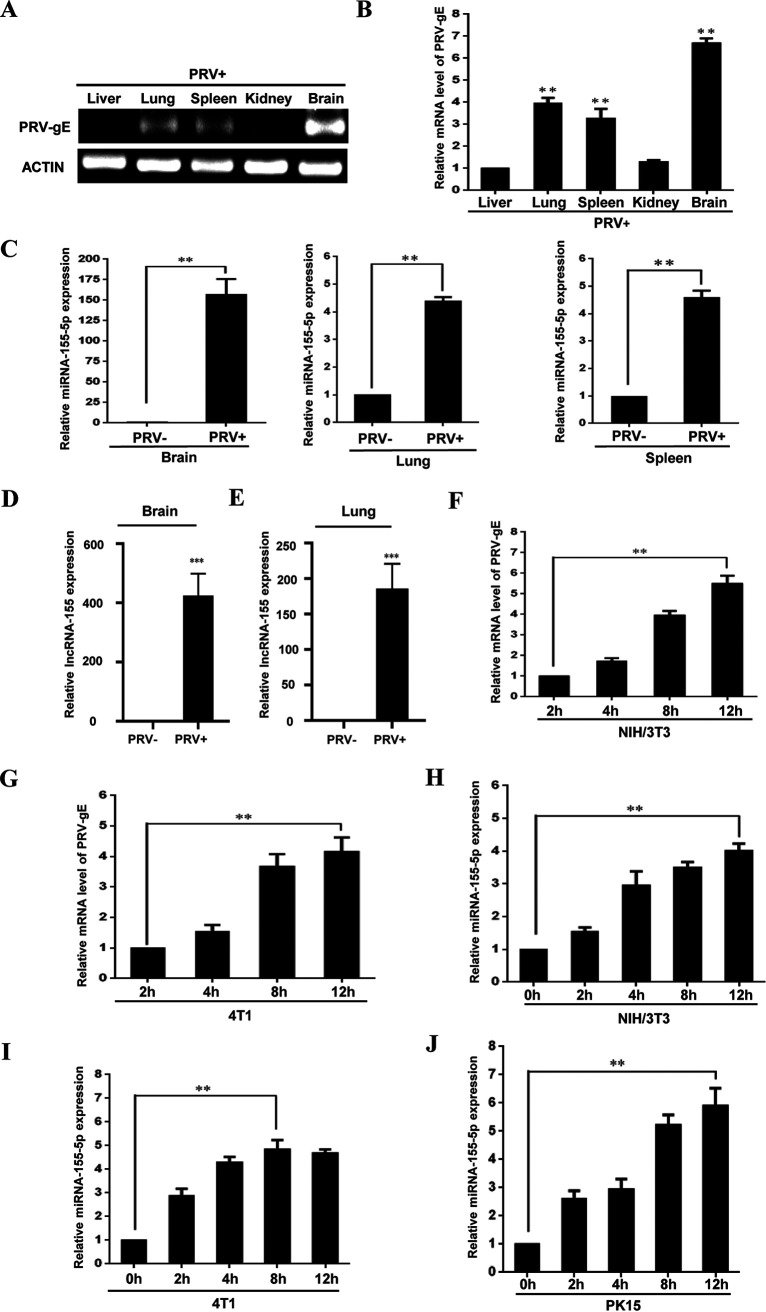
PRV infection induces robust expression of miRNA-155-5p and lncRNA-155. (A and B) The expression levels of PRV-gE in different mouse tissues (liver, lung, spleen, kidney, and brain) after PRV infection for 60 h were examined by RT-PCR (A) and qRT-PCR (B). (C to E) The expression levels of miRNA-155-5p (C) and lncRNA-155 (D and E) in the indicated mouse tissues infected with or without PRV were detected by qRT-PCR. (F and G) NIH/3T3 (F) and 4T1 (G) cells were infected with PRV for the indicated times. The mRNA levels of PRV-gE were examined by qRT-PCR. (H to J) NIH/3T3 (H), 4T1 (I), and PK15 (J) cells were infected with PRV for the indicated times, and qRT-PCR was performed to determine miRNA-155-5p expression in the cells. Data are shown as means ± SD; **, *P < *0.01; ***, *P < *0.001; *n* = 3.

Next, we asked whether lncRNA-155 and miRNA-155 have distinct effects on antiviral responses to DNA virus infection. For this, PRV, a DNA virus which can infect mice, was employed. Both lncRNA-155 and miRNA-155 have been characterized as inducible noncoding RNAs and regulators of immune responses (3, 5), but their involvement in the pathogenesis of many highly pathogenic viruses such as PRV, has not been explored. While there exist two mature forms of miRNA-155 species (miRNA-155-5p and miRNA-155-3p), miRNA-155-5p is the more abundant and functionally dominant miRNA-155 form (7). Thus, we used several mouse models and cell lines to examine the expression of miRNA-155-5p and lncRNA-155 following PRV infection. The expression of viral PRV-gE, a glycoprotein closely related to the virulence of PRV, was examined to indicate viral replication. As shown in Fig. 2A and B, expression of PRV-gE in several mouse tissues was verified after PRV infection for 60 h by RT-PCR and quantitative RT-PCR (qRT-PCR). Then, the virus-induced expression of lncRNA-155 and miRNA-155-5p was examined. The results showed that the expression of both lncRNA-155 and miRNA-155-5p was significantly augmented in several mouse tissues upon PRV infection (Fig. 2C to E and Fig. S2A and B). We further determined PRV replication kinetics in different PRV-infected mouse and swine cell lines at the indicated time points (Fig. 2F and G and Fig. S2C). Time course experiments showed that the expression of both miRNA-155-5p and lncRNA-155 was significantly increased with respect to the extent of viral replication in the indicated cells after PRV infection (Fig. 2H to J and Fig. S2D and E). Taken together, our observations indicate that PRV infection can induce the expression of both lncRNA-155 and miRNA-155-5p in mouse tissues and different cell lines.

10.1128/mbio.02510-22.2 FIG S2 PRV infection induces robust expression of lncRNA-155 both *in vitro* and *in vivo.* (A and B) Expression of lncRNA-155 in the indicated mouse tissues infected with PRV at the indicated time points was examined by RT-PCR. (C) PK15 cells were infected with PRV, and expression of PRV-gE was tested by RT-PCR at the indicated time points. (D and E) Time point experiments were performed to test the levels of lncRNA-155 expression in NIH/3T3 (D) and RAW 264.7 (E) cells at the indicated time points after PRV infection. Download FIG S2, TIF file, 0.4 MB. Copyright © 2022 Rai et al. 2022 Rai et al. https://creativecommons.org/licenses/by/4.0/ This content is distributed under the terms of the Creative Commons Attribution 4.0 International license.

### MiRNA-155-5p and lncRNA-155 suppress viral infection *in vitro* but to different extents.

**FIG 3:**
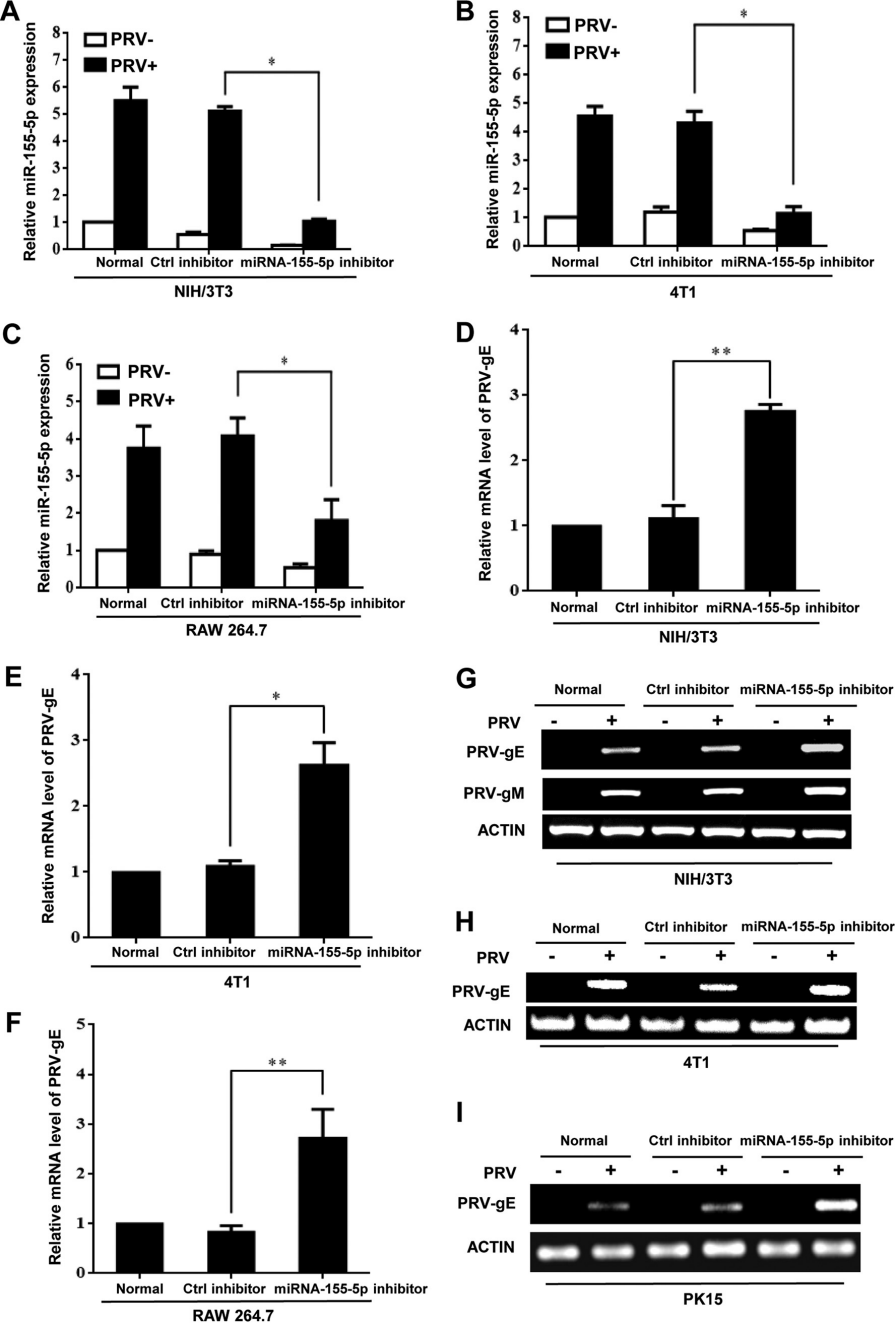
Depleting miRNA-155-5p promotes PRV replication *in vitro.* (A to C) NIH/3T3 (A), 4T1 (B), and RAW264.7 (C) cells were transfected with miRNA-155-5p inhibitor or control inhibitor and then infected with or without PRV for 12 h. qRT-PCR was performed to examine miRNA-155-5p expression. (D to F) The PRV-gE expression in NIH/3T3 (D), 4T1 (E), and RAW264.7 (F) cells transfected with miRNA-155-5p inhibitor or control inhibitor followed by PRV infection was detected by qRT-PCR. (G to I) NIH/3T3 (G), 4T1 (H), and PK15 (I) cells were transfected with miRNA-155-5p inhibitor or control inhibitor and then infected with PRV. The expression of PRV-gE and gM was examined by RT-PCR. Data are shown as means ± SD; *, *P < *0.05; **, *P < *0.01; *n* = 3.

To determine the role of miRNA-155-5p in PRV pathogenesis, both mouse and swine cell lines, including NIH/3T3, 4T1, RAW 264.7, and PK15 cells, were treated with miRNA-155-5p inhibitor, followed by PRV infection. The levels of miRNA-155-5p were detected by qRT-PCR analysis. As expected, treatment with miRNA-155-5p inhibitor significantly reduced the expression of miRNA-155-5p in these cells (Fig. 3A to C and Fig. S3A). Indeed, inhibition of miRNA-155-5p expression resulted in enhanced expression of PRV-gE compared to the controls in all the cell lines tested (Fig. 3D to I). Consistently, significantly enhanced expression of PRV-gM and higher viral titers were observed in miRNA-155-5p inhibitor-treated NIH/3T3 cells than in control cells (Fig. 3G and Fig. S3B and C). These results indicate that depleting miRNA-155-5p expression enhances PRV replication *in vitro*.

10.1128/mbio.02510-22.3 FIG S3 Depleting miRNA-155-5p promotes PRV replication *in vitro*. (A) PK15 cells were transfected with miRNA-155-5p inhibitor or the control and then infected with PRV. The miRNA-155-5p expression levels in the cells were examined by qRT-PCR. (B and C) NIH/3T3 was transfected with miRNA-155-5p inhibitor or control inhibitor and then infected with PRV for 12 h. qRT-PCR and plaque-forming assay were performed to examine the expression of PRV-gM (B) and viral titers (C). Data are represented as the mean ± SD. *, *P < *0.05; ***, *P < *0.001; *n* = 3. Download FIG S3, TIF file, 0.2 MB. Copyright © 2022 Rai et al. 2022 Rai et al. https://creativecommons.org/licenses/by/4.0/ This content is distributed under the terms of the Creative Commons Attribution 4.0 International license.

**FIG 4:**
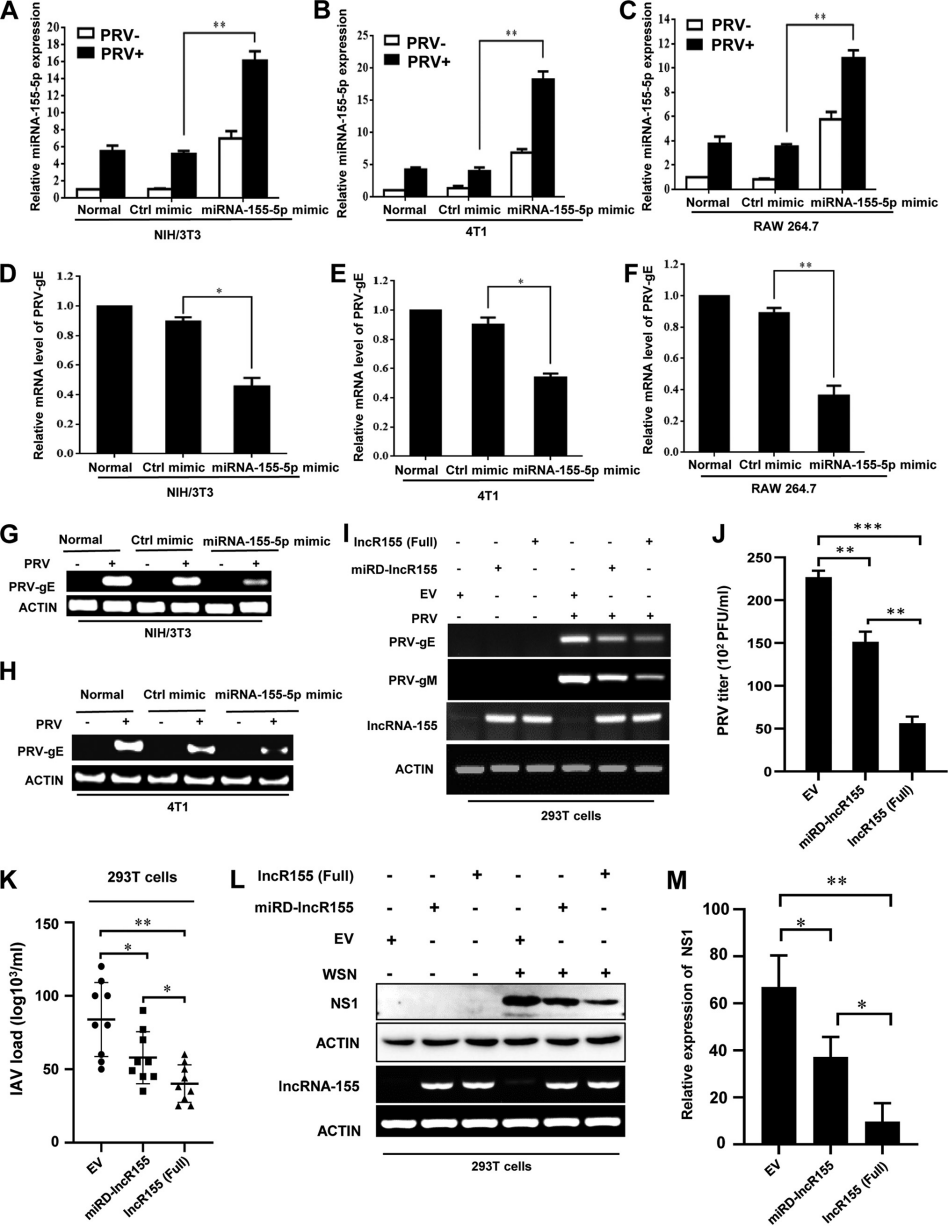
MiRNA-155-5p and lncRNA-155 suppress viral infection but to different extents. (A to C) NIH/3T3 (A), 4T1 (B), and RAW264.7 (C) cells were transfected with miRNA-155-5p mimic or control mimic and then infected with or without PRV for 12 h. qRT-PCR was performed to examine miRNA-155-5p expression levels. (D to H) The PRV-gE expression in NIH/3T3 (D and G), 4T1 (E and H), and RAW264.7 (F) cells transfected with miRNA-155-5p mimic or control mimic followed by PRV infection was detected by qRT-PCR (D to F) and RT-PCR (G and H). (I and J) 293T cells were transfected with full-length lncRNA-155 [lncR155 (Full)], miRNA-155-deleted lncRNA-155 (miRD-lncR155) or EV and then infected with PRV. The expression of PRV-gE and gM was examined by RT-PCR (I). Shown are representative data of three independent experiments with similar results. The PRV titers were examined by plaque-forming assay (J). (K and M) 293T cells were transfected with lncR155 (Full), miRD-lncR155, or EV and then infected with IAV. The IAV titers were examined by plaque-forming assay (K). The IAV NS1 protein levels were detected by Western blotting (L). Shown are representative immunoblots from three independent experiments with similar results. Relative levels of NS1 protein in panel L were quantitated by densitometry and normalized to actin levels (M). Data are shown as means ± SD; *, *P < *0.05; **, *P < *0.01; ***, *P < *0.001; *n* = 3.

On the other hand, we overexpressed miRNA-155-5p in NIH/3T3, 4T1, and RAW 264.7 cells by transient transfection with the miRNA-155-5p mimic followed by PRV infection. Levels of miRNA-155-5p overexpression were confirmed in the cells after transfection with the mimic and PRV infection (Fig. 4A to C). As expected, transfection with miRNA-155-5p mimic caused a significant decrease in the expression of PRV-gE/gM and viral titers in the indicated cells compared to transfection with the control as examined by qRT-PCR, RT-PCR, and plaque forming assay, respectively (Fig. 4D to H and Fig. S4A to C).

10.1128/mbio.02510-22.4 FIG S4 Overexpression of both lncR-155 and miRNA-155-5p suppresses viral infection *in vitro* but to different extents. (A to C) NIH/3T3 cells were transfected with miRNA-155-5p mimic or control mimic and then infected with or without PRV for 12 h. RT-PCR (A), qRT-PCR (B), and plaque-forming assay (C) were performed to examine expression of the indicated viral genes and viral titers from the supernatant of virus-infected cells. (D to F) siRNAs specifically targeting lncRNA-155 were used to disrupt the expression of lncRNA-155 in 293T cells, followed by infection with PRV for 12 h. The expression of lncRNA-155 and PRV-gE in lncRNA-155-depleted 293T cells was examined by RT-PCR and qRT-PCR, respectively. (G) Relative expression of lncRNA-155 in 293T cells transfected with plasmid encoding full length lncRNA-155 [lncR155 (Full)], miRNA-155-deleted lncRNA-155 (miRD-lncR155), or empty vector (EV) was quantified and normalized to actin levels (represented in Fig. 4I, *n* = 3). (H and I) 293T cells were transfected with lncR155 (Full), miRD-lncR155, or EV and then infected with PRV for 12 h. The relative expression of PRV-gE (H) and PRV-gM (I) was examined by qRT-PCR. (J) Relative levels of miRNA-155-5p in lncR155 (Full)-, or EV-transfected 293T cells with or without IAV infection were examined by qRT-PCR. (K and L) A549 (K) and 293T (L) cells overexpressing lncR155 (Full) or EV were generated, followed by treatment with miRNA-155-5p inhibitors and IAV infection. The expression of IAV NS1 was examined by RT-PCR. Data are represented as the mean ± SD. **, *P < *0.01; ***, *P < *0.001; *n* = 3. Download FIG S4, TIF file, 0.6 MB. Copyright © 2022 Rai et al. 2022 Rai et al. https://creativecommons.org/licenses/by/4.0/ This content is distributed under the terms of the Creative Commons Attribution 4.0 International license.

The results presented above indicated that miRNA-155-5p suppresses PRV replication *in vitro*. This prompted us to evaluate whether lncRNA-155 had any effects on PRV replication and whether there was any difference in function between lncRNA-155 with and without miRNA-155 sequence. For this, expression of lncRNA-155 was disrupted in 293T cells by transfecting specific small interfering RNAs (siRNAs) targeting lncRNA-155. The knockdown efficiency of lncRNA-155 was tested by RT-PCR and qRT-PCR (Fig. S4D and E). Depletion of lncRNA-155 caused significantly enhanced expression of PRV-gE (Fig. S4E and F). Then, we further addressed the involvement of miRNA-155 in the regulation of PRV replication by lncRNA-155. We constructed several vectors expressing either the full length lncRNA-155 [lncR155 (Full)], lncRNA-155 lacking the miRNA-155 sequence (miRD-lncR155), or empty vector (EV) control. 293T cells were then transfected with these plasmids, followed by infection with PRV (Fig. 4I and Fig. S4G). We observed that overexpression of either lncR155 (Full) or miRD-lncR155 caused a significantly diminished expression of PRV-gE and gM and reduced viral titers compared to the EV control. Interestingly, significantly decreased viral titers and lower expression of both PRV-gE and gM were observed in lncR155 (Full)-overexpressing cells compared with that in cells expressing miRD-lncR155 after PRV infection (Fig. 4I and J and Fig. S4H and I).

Next, we confirmed this finding by using the IAV infection model. Similar results were obtained by infection of IAV in these cells regarding viral titers and NS1 protein expression as detected by a plaque-forming assay and Western blotting, respectively. The IAV titers and NS1 protein levels were significantly reduced in lncR155 (Full)-overexpressing cells compared to the cells expressing either miRD-lncR155 or EV (Fig. 4K to M). Overexpression of miRD-lncR155 also had a significant effect on IAV replication compared with the EV control (Fig. 4K to M). To ensure that miRNA-155-5p can be expressed by lncR155 (Full), we examined the expression of miRNA-155-5p by qRT-PCR. Indeed, miRNA-155-5p was expressed in cells overexpressing lncR155 (Full) compared to the controls with or without WSN infection (Fig. S4J). Furthermore, we generated A549 and 293T cell lines overexpressing the full length of lncRNA-155 that were then treated with miRNA-155-5p inhibitor, followed by WSN infection. Notably, forced expression of lncR155 (Full) suppressed the expression of IAV NS1 in cells treated with or without miR155-5p inhibitor (Fig. S4K and L). Taken together, these data indicate that both lncRNA-155 and miRNA-155-5p suppress the replication of PRV and IAV, and full-length lncRNA-155 has a more profound inhibitory effect on the viral infection. In addition, the results reveal that lncRNA-155 without miRNA-155 sequence is functional in the antiviral response.

### LncRNA-155KO mice are also highly susceptible to PRV infection compared with miRNA-155KO mice.

**FIG 5:**
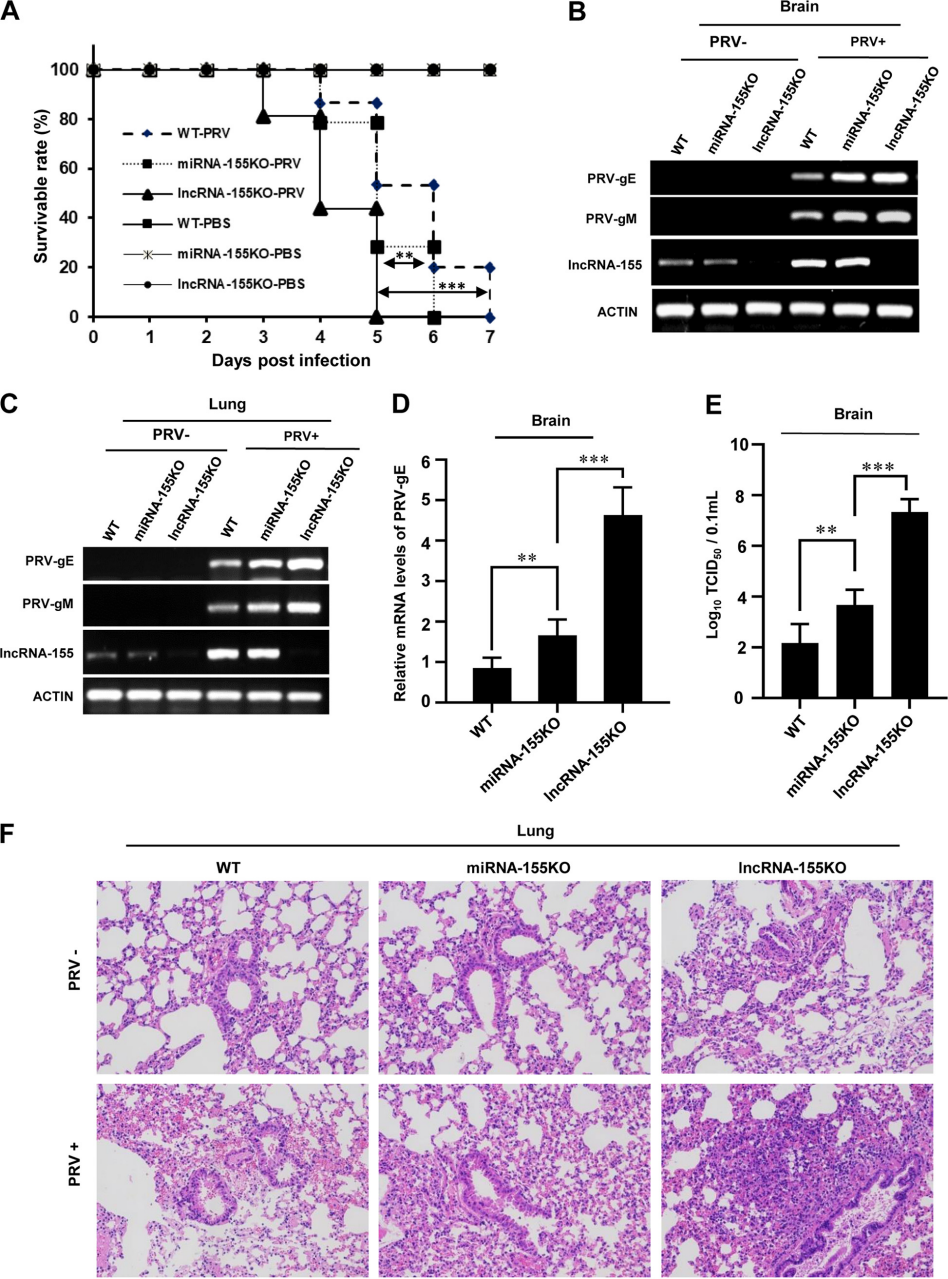
LncRNA-155KO mice are highly susceptible to PRV infection compared with miRNA-155KO mice. (A to F) WT, miRNA-155KO, and lncRNA-155KO mice (5 to 6 weeks old) were intramuscularly infected with PRV, and the effects of lncRNA-155 with or without miRNA-155 on the PRV virulence and infection kinetics in mice were determined by (A) survival rates (11 to 13 mice/group), (B to D) RT-PCR (B and C) and qRT-PCR (D) analysis of PRV-gE and gM expression in mouse brain and lung tissues at 2 dpi, (E) measuring viral loads in brain tissues employing the TCID_50_ assay at 2 dpi (3 to 5 mice/group), and (F) HE staining analysis of mouse lung tissues at 2 dpi (magnification ×200). The leukocyte infiltration was more pronounced in lncRNA-155KO mice than miRNA-155KO and WT mice. Data are shown as means ± SD; **, *P < *0.01; ***, *P < *0.001; *n* = 3.

Having found that PRV infection induced the expression of lncRNA-155 and miRNA-155-5p and that these noncoding RNAs affected PRV replication *in vitro*, we performed *in vivo* analysis in miRNA-155KO, lncRNA-155KO, and WT mice to investigate the involvement of miRNA-155 and lncRNA-155 in PRV pathogenesis. All three groups of mice (*n* = 14 to 16 per group) were infected with PRV intranasally and subjected to PRV infection susceptibility testing. Under our experimental conditions, lncRNA-155KO mice showed a significantly reduced survival rate compared to either miRNA-155KO or WT mice (Fig. 5A). The lncRNA-155KO mice started dying at 3 dpi and reached 100% mortality at 5 dpi, while the miRNA-155KO and WT mice began dying at 4 dpi, showing higher survival rates until 6 and 7 dpi, respectively (Fig. 5A). Next, we examined the expression of PRV-gE and -gM at 3 dpi by qRT-PCR and RT-PCR in the brain and lung tissues of the mice. In support of this, the expression of PRV-gE and -gM was higher in the brain and lung of lncRNA-155KO mice, followed by miRNA-155KO and WT mice infected with PRV (Fig. 5B to D and Fig. S5A to D). As determined by a 50% tissue culture infect dose (TCID_50_) assay, the virus titer in the brain tissues was also significantly higher in lncRNA-155KO mice than that in miRNA-155KO or WT mice infected with PRV (Fig. 5E and Fig. S5E). Moreover, HE staining of PRV-infected mouse lungs showed severe inflammation and more inflammatory cell infiltration in lncRNA-155KO mice than miRNA-155KO or WT mice (Fig. 5F). Additionally, the lncRNA-155KO mice exhibited severe pathological changes through analyses by HE staining and bioluminescence imaging, including cortical edema, neuronal contraction, and bleeding, compared to the WT control (Fig. S5F). These observations suggest that both lncRNA-155 and miRNA-155 are involved in antiviral immunity against PRV infection but to different extents.

10.1128/mbio.02510-22.5 FIG S5 LncRNA-155KO mice are highly susceptible to PRV infection compared with WT mice. (A) Shown are representative images of WT and lncRNA-155KO mice after PRV infection. (B) Expression of PRV-gM in the lung tissues of the indicated mice with or without PRV infection was examined by qRT-PCR. (C to F) WT and lncRNA-155KO mice were infected with PRV. PRV-gE expression in the brain tissues of the indicated mice with or without PRV infection was examined by RT-PCR (C) and qRT-PCR (D). The TCID_50_ values of PRV in mouse brain tissues were determined (E). Representative images of brain tissues from the indicated mice by HE staining (magnification, ×200) are shown in panel F. Data are represented as the mean ± SD. *, *P < *0.05; **, *P < *0.01; ***, *P < *0.001; *n* = 3. Download FIG S5, TIF file, 1.6 MB. Copyright © 2022 Rai et al. 2022 Rai et al. https://creativecommons.org/licenses/by/4.0/ This content is distributed under the terms of the Creative Commons Attribution 4.0 International license.

### Both miRNA-155 and lncRNA-155 can upregulate the phosphorylation of STAT1 during the viral infection.

**FIG 6:**
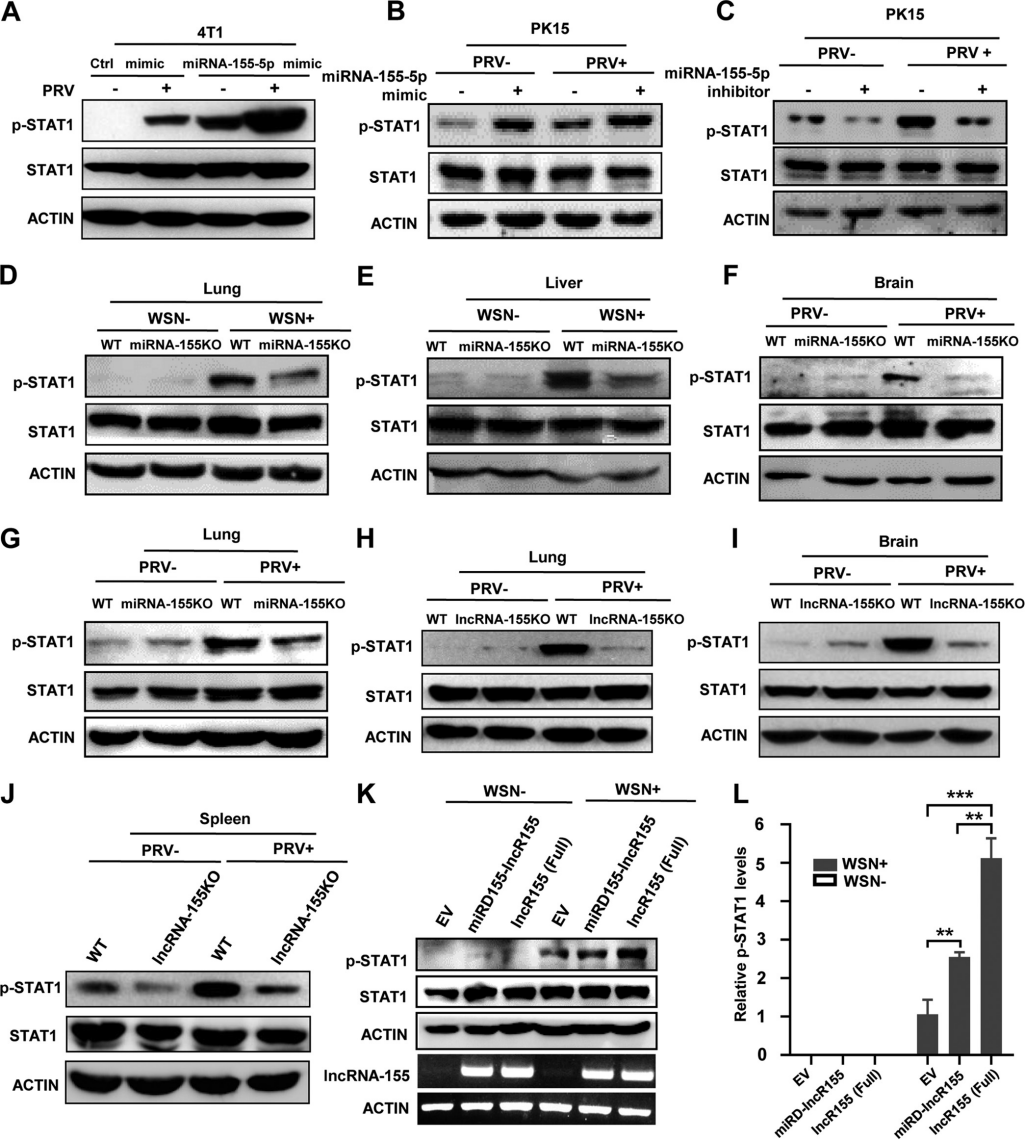
MiRNA-155 and lncRNA-155 enhance the phosphorylation of STAT1 during viral infection. (A and B) 4T1 (A) and PK15 (B) cells were transfected with miRNA-155-5p mimic or control mimic and then subjected to PRV infection for 12 h. STAT1 phosphorylation (p-STAT1) in the cells was examined by Western blotting. (C) PK15 cells were transfected with miRNA-155-5p inhibitor or control inhibitor followed by PRV infection for 12 h. Then, p-STAT1 was examined by Western blotting. (D and E) WT and miRNA-155KO mice were infected with WSN, and p-STAT1 in the indicated mouse tissues was tested by Western blotting. (F and G) WT and miRNA-155KO mice were infected with PRV, and p-STAT1 in indicated mouse tissues was tested by Western blotting. (H to J) WT and lncRNA-155KO mice were infected with PRV, and p-STAT1 in the indicated mouse tissues was detected by Western blotting. (K and L) 293T cells were transfected with lncR155 (Full), miRD-lncR155, or EV and then infected with WSN (MOI, 1) for 12 h. The levels of p-STAT1 were examined by Western blotting (K). Shown are representative immunoblots from three independent experiments with similar results. Relative levels of p-STAT1 in panel K were quantitated by densitometry and normalized to actin levels (L). Data are shown as means ± SD; **, *P < *0.01; ***, *P < *0.001; *n* = 3.

STAT1 plays a crucial role in the induction of antiviral responses (36). It has been recognized that SOCS1, a suppressor of JAK/STAT signaling, is one of the main targets of miRNA-155 (6, 7, 10). We therefore investigated whether miRNA-155 can regulate the phosphorylation of STAT1 (p-STAT1) during PRV and IAV infection. To this end, STAT1 activation was examined in 4T1 and PK15 cells transfected with miRNA-155-5p mimic with or without PRV infection (Fig. 6A and B and Fig. S6A). As predicted, miRNA-155-5p overexpression resulted in increased activation of STAT1 (p-STAT1 Y701) following viral infection compared to the control (Fig. 6A to B). Conversely, depletion of miRNA-155-5p using its inhibitor diminished virus-induced STAT1 activation in PK15 cells (Fig. 6C). For *in vivo* analysis, we examined the level of p-STAT1 in the indicated tissues of miRNA-155KO or WT mice upon IAV and PRV infection. The results showed that miRNA-155KO mice exhibited a lower level of p-STAT1 in the indicated tissues after the IAV infection (Fig. 6D and E). Similarly, the p-STAT1 level was clearly reduced in brain and lung of PRV-infected mice (Fig. 6F and G).

10.1128/mbio.02510-22.6 FIG S6 Knockout of lncRNA-155 greatly impairs PRV-induced phosphorylation of STAT1. (A) PK15 cells were transfected with miRNA-155-5p mimic or the control, followed by PRV infection. Expression of miRNA-155-5p in the cells was tested by qRT-PCR. (B to D) WT and lncRNA-155KO mice were intramuscularly infected with PRV for 60 h. The levels of p-STAT1 in the indicated mouse tissues were detected by Western blotting. Image J was used to quantify relative levels of p-STAT1 and then normalized to actin levels (represented in Fig. 6H to J). Data are represented as the mean ± SD. **, *P < *0.01; *n* = 3. Download FIG S6, TIF file, 0.2 MB. Copyright © 2022 Rai et al. 2022 Rai et al. https://creativecommons.org/licenses/by/4.0/ This content is distributed under the terms of the Creative Commons Attribution 4.0 International license.

Our previous study showed that overexpression of lncRNA-155 enhances activation of STAT1 induced by IAV (3). Next, we determined whether lncRNA-155 had any effects on PRV-induced STAT1 activation. To this end, we compared the virus-induced activation of STAT1 between lncRNA-155KO and WT mice infected with PRV. Remarkably, lncRNA-155KO mice demonstrated a significantly lower level of p-STAT1 in several mouse tissues in response to PRV infection (Fig. 6H to J and Fig. S6B to D). To compare the effects of lncRNA-155 and miRD-lncR155 on virus-induced activation of STAT1, we further examined the activation of STAT1 in miRD-lncR155- or full length lncRNA155-overexpressed 293T cells after WSN infection. Overexpression of miRD-lncR155 caused significantly higher activation of STAT1 than that of the EV control following IAV infection. Of interest, overexpression of full-length lncRNA-155 resulted in the highest activation of STAT1 after virus infection (Fig. 6K and L). Collectively, these data suggest that both lncRNA-155 and miRNA-155 can enhance the virus-induced activation of STAT1.

### Distinct layers of antiviral responses are modulated by miRNA-155 and lncRNA-155.

It has been shown that miRNA-155 potentiates the IFN signaling but may not affect IFN-β production (10). We also observed that an miRNA-155 mimic only slightly inhibited the virus-induced expression of IFN-β (Fig. S7A). However, overexpression of lncRNA-155 without miRNA-155 has been found to augment IFN-β production after virus infection (3). These data suggested a distinct layer of innate immune regulation mediated by miRNA-155 and lncRNA-155.

**FIG 7:**
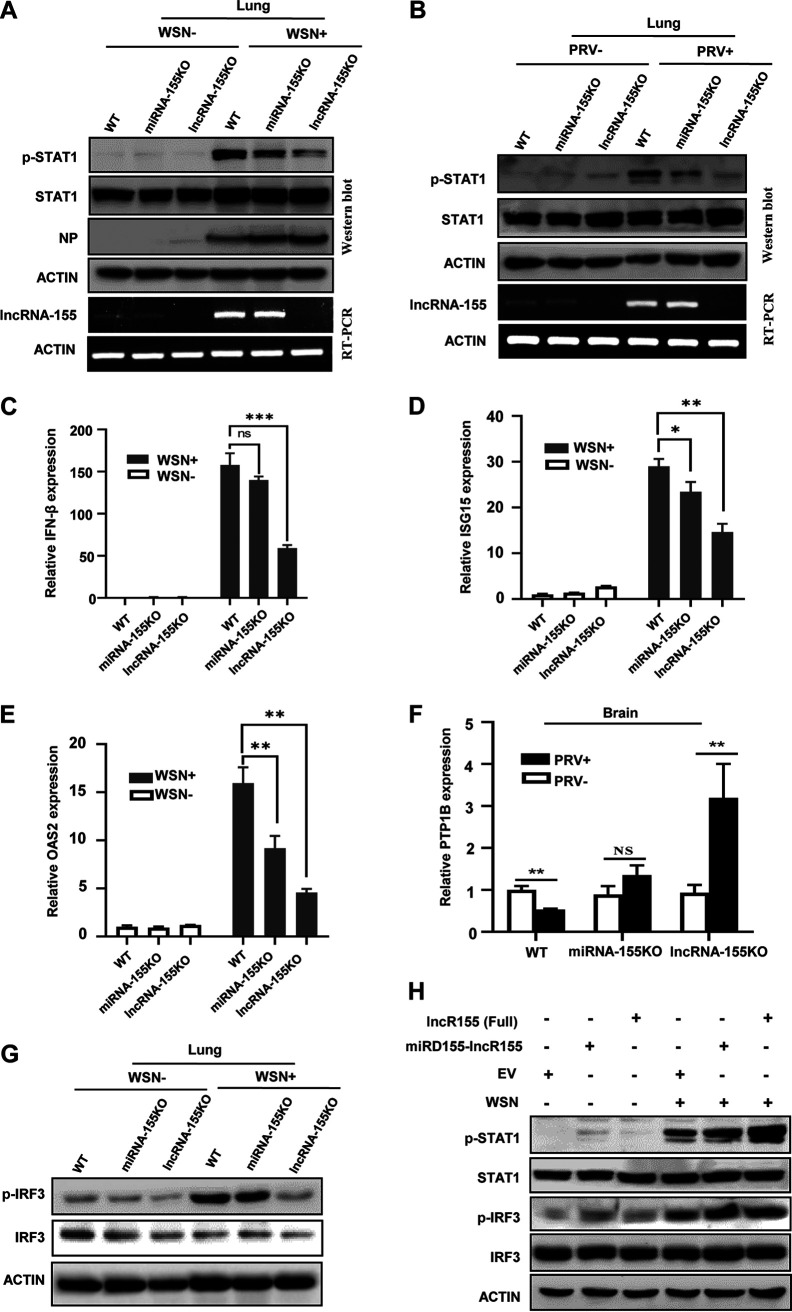
Distinct layers of antiviral responses are modulated by miRNA-155 and lncRNA-155. (A to F) WT, miRNA-155KO, and lncRNA-155KO mice were infected with IAV (A and C to E) or PRV (B and F). IAV infection-induced p-STAT1 (A) and expression of lncRNA-155 (A and B), IFN-β (C), ISG15 (D), or OAS2 (E) in mouse lungs were examined by Western blotting, RT-PCR, and qRT-PCR respectively. PRV infection-induced p-STAT1 (B) and expression of PTP1B (F) in the indicated mouse tissues were detected by Western blotting and qRT-PCR, respectively. (G) WT, miRNA-155KO, and lncRNA-155KO mice were infected with IAV. The levels of p-IRF3 in mouse lungs were examined by Western blotting. Shown are representative immunoblots from three independent experiments with similar results. (H) 293T cells were transfected with lncRNA-155 (Full), miRD-lncRNA-155, or EV and then infected with WSN (MOI, 1) for 12 h. The levels of p-STAT1 and p-IRF3 were tested by Western blotting. Shown are representative immunoblots from three independent experiments with similar results. Data are shown as means ± SD; *, *P < *0.05; **, *P < *0.01; ***, *P < *0.001; *n* = 3.

10.1128/mbio.02510-22.7 FIG S7 Distinct layers of antiviral responses are modulated by miRNA-155 and lncRNA-155. (A) PK15 cells were transfected with miRNA-155-5p mimic or the controls and then infected with or without PRV for 10 h. The expression of IFN-β was examined by qRT-PCR. Shown are representative data from three experiments with similar results. (B to E) WT, miRNA-155KO, and lncRNA-155KO mice were infected with IAV for 3 dpi. The levels of p-STAT1 (B to D) and p-p65 (E) in the indicated mouse tissues were examined by Western blotting. (F) Relative levels of p-IRF3 (represented in Fig. 7G, *n* = 3) in the indicated mice were quantitated by densitometric analysis using ImageJ and normalized to actin. (G and H) Relative levels of p-STAT1 (G) and p-IRF3 (H) (represented in Fig. 7H, *n* = 3) in the cells were quantitated by densitometric analysis using ImageJ and normalized to actin levels. Data are represented as the mean ± SD. **, *P < *0.01; ***, *P < *0.001; *n* = 3. Download FIG S7, TIF file, 0.5 MB. Copyright © 2022 Rai et al. 2022 Rai et al. https://creativecommons.org/licenses/by/4.0/ This content is distributed under the terms of the Creative Commons Attribution 4.0 International license.

To elucidate the distinct roles of miRNA-155 and lncRNA-155 in regulating antiviral immune signaling *in vivo*, we utilized three groups of mice: lncRNA-155KO, miRNA-155KO, and WT. The results showed that lncRNA-155KO mice exhibited the lowest level of p-STAT1, followed by miRNA-155KO and WT mice, after IAV infection in various mouse tissues (Fig. 7A and Fig. S7B to D). Similar results were obtained from observations of the lungs of mice infected with PRV (Fig. 7B). Next, we compared the virus-induced expression of IFN-β and production of important ISGs by qRT-PCR. Interestingly, virus-induced expression of IFN-β was significantly decreased in lncRNA-155KO mice compared with their WT counterparts (Fig. 7C). However, there were no significant differences in IFN-β production between miRNA-155KO mice and WT control mice after IAV infection (Fig. 7C). Of note, expression of critical ISGs in miRNA-155KO mice was significantly lower than that in WT mice, and the lowest levels of these ISGs were observed in lncRNA-155KO mice (Fig. 7D and E), despite no significant differences in virus-induced IFN-β production between miRNA-155KO and WT mice (Fig. 7C).

Our previous study demonstrated that lncRNA-155 augments IFN-β production by inhibiting the expression of protein-tyrosine phosphatase 1B (PTP1B) in cells infected with IAV (3). Here, we found that the expression of PTP1B was significantly decreased in WT mice, slightly increased, but not significantly, in miRNA-155KO mice, and very significantly increased in lncRNA-155KO mice after the PRV infection (Fig. 7F). Since PTP1B can target the IRF-IFN-stimulated response elements (ISRE) pathway (37), we further examined the virus-induced activation of antiviral immune signaling upstream of IFN production in these animals. The results showed that lncRNA-155KO mice exhibited a dramatic impairment of IRF3 and p65 activation, but miRNA-155KO mice displayed only a slightly diminished activation of IRF3 compared to WT mice after IAV infection (Fig. 7G and Fig. S7E and F). Finally, we examined virus-induced activation of innate immune signaling in 293T cells overexpressing either miRNA-155-deleted lncRNA-155 (miRD-lncR155), full-length lncRNA-155 or empty vector (EV) control. In response to IAV infection, overexpression of either miRD-lncR155 or full-length lncRNA-155 increased the level of p-STAT1, and the latter appeared to be more potent than the miRD-lncR155 (Fig. 7H and Fig. S7G). However, no obvious difference was observed in the level of virus-induced p-IRF3 between two groups overexpressing either full-length lncRNA-155 or miRD-lncR155 (Fig. 7H and Fig. S7H). These results suggest that miRNA-155 may mainly have an effect downstream of IFN production, while lncRNA-155 may positively regulate innate immune signaling that governs IFN expression. Together, our data indicate that lncRNA-155 and miRNA-155 play distinct roles in regulating innate antiviral responses.

## DISCUSSION

Increasing evidence has unraveled the diversified biological functions of both lncRNAs and miRNAs, including their specific roles in regulating innate antiviral immunity. Recently, we identified lncRNA-155 as a novel positive regulator of innate immunity (3). In the previous study, we also employed lncRNA-155KO mice in which most of the lncRNA-155 sequences are deleted along with pre-miR155. However, the limitation of these experiments using the lncRNA-155KO mice was that they could not compare functional differences between lncRNA-155 without miRNA-155 and miRNA-155 alone *in vivo*. To provide more conclusive insights into the functions of *MIR155HG*, we generated miRNA-155KO mice that carry a 19-bp deletion targeting the pre-miRNA-155 sequence. The 65-nucleotide (nt) pre-miR155 is produced in Mus musculus through hydrolysis of the lncRNA-155 by Drosha RNase III. Pre-miRNA-155 is then transported to the cytoplasm, forming a stem-loop structure to be further cleaved by RNase III enzyme Dicer at the terminal loop, giving rise to the 22-nt miRNA duplex, called miRNA-155 (2). The deletion of 19 bp causes the most minimal distortion of the lncRNA structure while deforming the stem-loop structure of pre-miRNA-155, which disables the Dicer from terminal loop recognition and prevents pre-miRNA-155 from processing into mature miRNA-155 species and also deletes three critically functional nucleotides in the mature miRNA-155 sequences. Our experiments demonstrate that mature miRNA-155 is not expressed in miRNA-155KO mice, while lncRNA-155 with a deletion of miRNA-155 sequence is expressed normally in these animals. Thus, we used three groups of mice (lncRNA-155KO, miRNA-155KO, and WT), to determine the distinct functional roles of lncRNA-155 and miRNA-155 in regulating innate antiviral immunity *in vivo*.

In our *in vivo* analysis, the survival rates of lncRNA-155KO mice were clearly lower than those of miRNA-155KO and WT mice following IAV infection, but the difference was not statistically significant. Indeed, statistical significance and biological relevance may not be used interchangeably in survival studies. A cutoff *P* value of 0.05 is relative, but if the *P* value is more than 0.05, the outcome can still reflect a biologically important effect (38). In order to determine different effects of lncRNA-155 and miRNA-155 on IAV pathogenesis, a low dose of IAV was used in this study since lncRNA-155KO mice are highly sensitive to IAV infection. A clear difference in survival rates was observed between lncRNA-155KO and miRNA-155KO or WT mice, as more than 60% of WT mice and approximately 55% of miRNA-155KO mice survived even beyond 10 dpi, but only 27.27% of lncRNA-155KO mice survived at this time point. In our previous study, all lncRNA-155KO mice died within 8 dpi when a higher dose of the virus was used (3). Additionally, our present results showed that lncRNA-155KO mice showed a significantly reduced survival rate compared to either miRNA-155KO or WT mice following PRV infection, during which all lncRNA-155KO mice died within 5 dpi. Overall, our *in vivo* results showed that lncRNA-155KO mice were more susceptible to the viral infection than miRNA-155KO mice, suggesting that lncRNA-155 has an additional role in antiviral immunity besides processing miRNA-155.

It is well known that miRNA-155 plays an essential role in various biological processes such as tumor formation, inflammatory response, immunity, and viral pathogenesis (5, 9, 10). Previous studies have shown that those viral infections can induce the expression of miRNA-155, and the inducible miRNA-155 is involved in regulating virus-host interaction (9, 10, 39–41). However, the functional involvement of miRNA-155 in the pathogenesis of PRV was poorly understood. Here, we investigated the effect of miRNA-155 on PRV infection. In concordance with most of the previous reports on other viruses (41), PRV infection also induced the expression of miRNA-155-5p in different cell lines *in vitro* and various mouse tissues *in vivo.* Moreover, we found that lncRNA-155 was also inducible by PRV infection. Thus, the *in vivo* PRV susceptibility was tested among the WT, miRNA-155KO, and lncRNA-155KO mice. The results showed that lncRNA-155KO mice were the most susceptible among these groups, followed by the miRNA-155KO mice and then WT mice, suggesting that lncRNA-155 and miRNA-155 have independent functions and act cooperatively in the regulation of PRV infection *in vivo.* To provide more supportive evidence for the role of miRNA-155 alone in PRV pathogenesis, we performed *in vitro* functional experiments in several cell lines. Indeed, overexpression of miRNA-155-5p significantly inhibited PRV replication, while knockdown of miRNA-155-5p enhanced viral replication. It has been widely reported that miRNA-155 functions as an antiviral factor against several viruses, such as IAV (29), hepatitis B virus (32), West Nile virus (42), and infectious bursal disease virus (40). To some extent, however, proviral functions of miRNA-155 have also been identified (41, 43, 44). Exploring the molecular mechanism underlying miRNA-155 mediated antiviral and pro-viral effects remains a challenge. It has been speculated that optimal regulation of miRNA-155 expression is critical for exerting its effective antiviral immune response; dysregulation of miRNA-155 may result in disruption of the normal response to viral infection, leading to failure in the viral clearance (41).

Pre-miRNA-155 gives rise to two mature miRNAs, miRNA-155-5p and miRNA-155-3p. Of them, miRNA-155-5p is the more abundant and functionally dominant form (7, 45). It has been shown that miRNA-155-5p inhibits the expression of IFN-α/β by targeting mRNA of TGF-β-activated kinase 1/MAP3K7-binding protein 2 (TAB2) for degradation, while miRNA-155-3p targets interleukin-1 receptor-associated kinase 3 (IRAK3) mRNA, leading to the upregulation of IFN-α/β (7). Many studies have suggested that miRNA-155 enhances IFN-β expression. In contrast, other studies indicate that miRNA-155 inhibits IFN-β expression through inhibiting toll-like receptor (TLR3)-dependent signaling (7, 46). These discrepancies about the function of miRNA-155 might be due to the different experimental conditions used, such as different host species, virus types, and cell types. Here, we found that depletion of miRNA-155-5p resulted in diminished activation of STAT1, and overexpression of miRNA-155-5p caused a dramatic activation of STAT1 following PRV infection in several cell lines. In particular, we validated the function of miRNA-155-5p in response to PRV infection in PK15 cells. We found that neither the miRNA-155-5p mimic nor the miRNA-155-5p inhibitor profoundly affected the IFN-β production in PK15 cells. The results imply that miRNA-155 may regulate innate immunity by mediating the downstream of IFN (e.g., STAT1 activation) rather than inducing IFN-β production. Indeed, it has been shown that SOCS1, a downstream signaling molecule of IFN, is the main target of miRNA-155 (9, 10, 29), leading to enhanced phosphorylation of STAT1 (47).

On the other hand, our previous study revealed that lncRNA-155 inhibits the expression of PTP1B *in vitro* (3). In mice, PTP1B has been identified as a potent negative regulator of the IRF-ISRE-IFN-β pathway (37). Therefore, we examined the virus-induced activation of antiviral immune signaling upstream of IFN production. Interestingly, we found that lncRNA-155KO mice exhibited a dramatic impairment of IRF3 and NF-κB (p65) activation, but miRNA-155KO mice showed slightly diminished or even comparable activation of IRF3 and p56 compared to WT mice after IAV infection. Unlike previous *in vivo* studies using lncRNA-155KO mice that are deficient in miRNA-155 and lncRNA-155 (3), this research employed an additional group of miRNA-155KO mice, which specifically prevents the formation of both mature miRNA-155-5p and miRNA-155-3p but keeps the functional lncRNA-155. Therefore, the phenotypes observed in miRNA-155KO mice represent the function of lncRNA-155 without miR155. Compared to miRNA-155KO or WT mice, lncRNA-155KO mice displayed a significantly reduced production of IFN-β; compared to WT mice, miRNA-155KO mice did not show a significant difference in IFN-β production after IAV infection. However, the expression levels of several critical ISGs were significantly lower in miRNA-155KO than in WT mice, and the lowest expression of ISGs occurred in lncRNA-155KO mice. The results suggest that virus-induced lncRNA-155 provides a positive regulation for IFN-β production. Using overexpression of miRD-lncR155 and full-length lncRNA-155 in 293T cells, we found that IRF3 activity was significantly increased in lncRNA-155-expressing cells; there was no significant difference in virus-induced activation of IRF3 between the cells expressing full-length lncRNA-155 or miRD-lncR155. The results suggest that miRNA-155 has little effect on IRF3 and its upstream signaling. Together, our findings demonstrate that lncRNA-155 and miRNA-155 have distinct roles and act cooperatively in positively regulating antiviral responses through different mechanisms.

In summary, we observed that several viruses could induce the robust expression of lncRNA-155 and miRNA-155 in various cell lines and animal models, and lncRNA-155 without processing miRNA-155 promotes innate immunity. The results from our miRNA-155KO and lncRNA-155KO mouse models reveal that lncRNA-155 and miRNA-155 have distinct roles and act cooperatively in the regulation of innate immunity. This research provides a better understanding of the bivalent role of *MIR155HG* in regulating antiviral responses.

## MATERIALS AND METHODS

### Ethics statement.

The animal care and the protocols used in this study were approved by the Regulation of College of Animal Sciences, Fujian Agriculture and Forestry University of Research Ethics Committee (permit number PZCASFAFU2019002). All mouse experiments were carried out according to the Regulations for the Administration of Affairs Concerning Experimental Animals approved by the State Council of People’s Republic of China.

### Viruses and cells.

Influenza virus strain A/WSN/33 (H1N1) and PRV strain Min-A were propagated in specific-pathogen-free chicken embryos and Madin-Darby canine kidney (MDCK) cells, respectively, as previously described (48). For viral infection, cells were washed with phosphate-buffered saline (PBS) and infected with PRV at a dose of 1 × 10^4^ TCID_50_ or with the indicated multiplicity of infection (MOI) of IAV in Dulbecco’s modified Eagle’s medium (DMEM) containing 2 μg/mL l-1-tosylamido-2-phenylethyl chloromethyl ketone (TPCK)-treated trypsin, 100 U/mL penicillin, and 100 μg/mL streptomycin for 45 min at 37°C. After adsorption, the used medium was aspirated, and the cells were cultured with fresh DMEM for the indicated time.

Mouse embryo fibroblast cells (NIH/3T3), mouse Abelson murine leukemia virus-transformed macrophages (RAW 264.7), mouse breast cancer cells (4T1), MDCK cells, porcine kidney cells (PK15), and human embryonic kidney cells (HEK293T/293T) were obtained from the American Type Culture Collection (ATCC) (Manassas, VA, USA) and cultured in DMEM with 10% heat-inactivated fetal bovine serum (FBS) (Gibco, USA), 2 mM glutamine, 100 U/mL penicillin, and 100 μg/mL streptomycin. To establish miRNA-155 overexpression or knockdown cells, the oligonucleotide mus-miRNA-155-5p mimic, mus-miRNA-155-5p inhibitor, sus-miRNA-155-5p mimic, and sus-miRNA-155-5p inhibitor were chemically synthesized by RiboBio (Guangzhou, China). These synthetic oligonucleotides were transiently transfected into the NIH/3T3, RAW 264.7, 4T1, and PK15 cells in 6-well plates at a final concentration of 100 nM using Lipofectamine 2000 (Invitrogen, Carlsbad, CA, USA). The specific siRNA targeting lncRNA-155 and the control siRNA used in this study were chemically synthesized by RiboBio (Guangzhou, China). The plasmids expressing lncRNA-155 or miRNA-155-deleted lncRNA-155 (3) were used and expressed in 293T cells as previously described (3, 49).

### Generation of miRNA-155KO mice.

MiRNA-155KO mice (lacking only 19 bp of the miRNA-155 core sequence) on C57BL/6J background were generated using CRISPR/Cas9 by Bcgen (Beijing Biocytogen, Beijing, China). In brief, single guide RNAs (sgRNAs) were designed to target upstream and downstream introns of exon 2 of the gene MIR155HG located in chromosome 16. The sequences of screened guide RNAs with high on-target activity were as follows: upstream 5′-TATTCTGACGTACATCCCACAGG-3′, downstream 5′-GATTTGCCTAAGCATGCAGTAGG-3′. The constructed targeting vector contains exon 2 of the gene MIR155HG introducing a 19-bp deletion of the miRNA-155 core sequence (GGTTTTGGCCTCTGACTGACT>GT) and 2 left (1,400 bp) and right (1,400 bp) homology arms. The targeting vector and *in vitro*-synthesized sgRNAs and Cas9 mRNA were then coinjected into C57BL/6J mouse zygotes. After injection, the surviving zygotes were transferred into pseudopregnant mice. Knockout of miRNA-155 was examined by sequencing and qRT-PCR analysis. The miRNA155KO mice were physically fit, viable, and fertile with normal behavior; no gross or microscopic pathological changes that could influence the PRV/IAV challenge experiment were observed.

### Animal experiments.

Wild type (WT) C57BL/6J mice (5 to 6 weeks old, 18 to 20 g) were obtained from Vital River Laboratory Animal Center (Beijing, China). B6.Cg-miRNA-155tm1.1^Rsky^/J KO mice on C57BL/6J background (here called lncRNA-155KO mice) were purchased from the Jackson Laboratory (KO mice, stock 007745) (50). The KO mice were physically fit, viable, and fertile with normal behavior; no gross or microscopic pathological changes that could influence the PRV challenge experiment were observed. For PRV infection experiments, the mice were injected intramuscularly with PRV at a dose of 5 × 10^4^ TCID_50_. After injection for the indicated time, mice were euthanized, and their organs were collected for further experiments and analysis. Animal infection with IAV was performed as described previously (3).

### RNA preparation, RT-PCR, and qRT-PCR.

Total RNA was extracted from cells or tissues using TRIzol reagent (Tiangen, Beijing, China). Then, 2 μg of total RNA and Moloney murine leukemia virus (M-MLV) reverse transcriptase (Promega, Madison, WI, USA) were used to prepare cDNA. After cDNA synthesis, *Taq* DNA polymerase (TaKaRa Bio, Inc., Tokyo, Japan) was used in RT-PCR, and a SYBR fast qPCR kit (Kapa Biosystems, Inc., Wilmington, MA, USA) was employed in qRT-PCR. MicroRNAs were collected with an RNAmisi miRNA isolation kit (Aidlab, Beijing, China). Then, an miRNA real-time PCR assay kit (Aidlab, Beijing, China) was used for cDNA synthesis according to the manufacturer’s instructions. The primers used in this study were mouse miRNA-155-5p-forward (5′-TTAATGCTAATTGTGATAGGGGT-3′), hsa-miRNA-155-5p-forward (5′-TTAATGCTAATTGTGATAGGGGT-3′), and porcine miRNA-155-5p-forward (5′-TTAATGCTAATTGTGATAGGGGT-3′). U6 small nuclear RNA (snRNA) was used to normalize the expression of microRNAs. Other primers used in this study are listed in Table S1.

10.1128/mbio.02510-22.8 TABLE S1 Sequences of primers used in RT-PCR and qRT-PCR. Download Table S1, DOCX file, 0.01 MB. Copyright © 2022 Rai et al. 2022 Rai et al. https://creativecommons.org/licenses/by/4.0/ This content is distributed under the terms of the Creative Commons Attribution 4.0 International license.

### Western blotting.

Cells were lysed in radioimmunoprecipitation assay (RIPA) lysis buffer (50 mM Tris-HCl, pH 7.4, 150 mM NaCl, NP-40, 1 mM EDTA, and 1× protease inhibitor cocktail [Roche, Mannheim, Germany]) according to the manufacturer’s protocol. The samples were separated with sodium dodecyl sulfate (SDS)-polyacrylamide gel electrophoresis, transferred onto nitrocellulose membrane, and probed with the indicated antibodies. Where indicated, Western blot signals were quantified by densitometry as previously described (51, 52). The following antibodies were used: anti-polyclonal-NP (produced in our lab), anti-phospho-IRF3 (Abcam, Cambridge, UK), anti-IRF3 (Proteintech, Wuhan, China), anti-STAT1 (Abcam), anti-phospho-STAT1 (Abcam), anti-β-actin (TranGens Biotech, Beijing, China), anti-p65 (Abclonal, Wuhan, China) and anti-phospho-p65 (Abclonal).

### TCID_50_ and plaque-forming assay.

The mice were infected with the PRV Min-A strain, and the brain tissues were dissected and ground on the indicated days postinfection. Then, the supernatants were collected and diluted multiple times with the cell maintenance medium. The diluted supernatants were used to infect PK15 cells cultured in 96-well plates (100 mL/well). The last column of the 96-well plate was added with the cell maintenance medium (100 mL/well) as a negative control. The cells were incubated with the supernatants at various dilutions in a 37°C, 5% CO_2_ incubator. Cytopathic effect (CPE) was continuously monitored, and the number of CPE was calculated. The TCID_50_ of the virus was calculated according to the Reed-Muench method (53). For the plaque-forming assay, serial dilutions of the viral culture supernatants of the indicated cells infected with PRV were added to 6-well plates with a confluent monolayer of PK15 cells. The plates were then incubated at 37°C with gentle agitation at every 20 min interval. The plates were washed three times with PBS to remove excess virus inocula. Then, 2 mL overlay medium (2% methyl cellulose with DMEM medium containing 2% FBS) was added to PRV-infected PK15 cells and incubated at 37°C with 5% CO_2_ for 2 to 3 days. The cells were then stained with 0.5% crystal violet, and the plaques were counted.

### Statistical analysis.

Student’s *t* test was used to analyze the statistical difference. Data are represented as the mean ± standard deviation (SD), with a significant difference set at *P < *0.05. Survival curves were analyzed with Prism X7 using the log-rank test (GraphPad Software, Inc., San Diego, CA, USA).
