# Supplementary material for: MIR155HG Plays a Bivalent Role in Regulating Innate Antiviral Immunity by Encoding Long Noncoding RNA-155 and microRNA-155-5p
Source: mBio. 2022 Nov 2;13(6):e02510-22. doi: 10.1128/mbio.02510-22 (PMC9765511; doi:10.1128/mbio.02510-22)
Supplement: TABLE S1 [file mbio.02510-22-s0008.docx]

| **Mouse Primers** | |
| --- | --- |
| m-Actin-F | 5’ CATTGCTGACAGGATGCAGAAGG |
| m-Actin-R | 5’ TGCTGGAAGGTGGACAGTGAGG |
| m-lncRNA-155-F1 (for RT-PCR) | 5’ TACTATCAGTGCTGCAAACCAGG |
| m-lncRNA-155-R1 (for RT-PCR) | 5’ ACATGTGGGCTTGAAGTTGAGA |
| m-lncRNA-155-F2 (for RT-qPCR) | 5’ ACCAGCTCATCTGAGAAAACA |
| m-lncRNA-155-R2 (for RT-qPCR) | 5’ CAGGTAGGAGTCAGTCAGAGG |
| m-IFNβ-F | 5’ GCCTTTGCCATCCAAGAGATGC |
| m-IFNβ-R | 5’ ACACTGTCTGCTGGTGGAGTTC |
| m-OAS2-F | 5’ CCGGGCCAGTGCACAAGTTAG |
| m-OAS2-R | 5’ CGATGGCACCGAGGACACC |
| m-ISG15-F | 5’ AGCAAGCAGCCAGAAGCAGACTC |
| m-ISG15-R | 5’ GGAAAGCCGGCACACCAATC |
| m-PTP1B-F | 5’ CATCAAGAAAGTACTGCTGGAGATGC |
| m-PTP1B-R | 5’ TCCTTCCACTGATCCTGCACTGAC |
| **Human Primers** | |
| h-Actin-F | 5’ CACCATTGGCAATGAGCGGTTC |
| h-Actin-R | 5’ AGGTCTTTGCGGATGTCCACGT |
| h-lncRNA-155-F | 5’ CCGTGGGAGGATGACAAAGA |
| h-lncRNA-155-R | 5’ CGTTACCTGGGGGAAAGTACC |
| **Sus Primers** | |
| sus-Actin-F | 5’ TCTGGCACCACACCTTCT |
| sus-Actin-R | 5’ TGATCTGGGTCATCTTCTCAC |
| sus-IFNβ-F | 5’ CATCCTCCAAATCGCTCTCC |
| sus-IFNβ-R | 5’ CTGACATGCCAAATTGCTGC |
| **Other Primers** | |
| PRV-gE-F | 5’ CTTCCACTCGCAGCTCTTCT |
| PRV-gE-R | 5’ TAGATGCAGGGCTCGTACAC |
| PRV-gM-F | 5’ CTGGCTGATAGAGGTCTGCGGGTTCG |
| PRV-gM-R | 5’ CCGGAGGTGTCGTTGAGCGTGTCGT |

**Supplementary Table 1**
